# Association of sleep with emotional and behavioral problems among abused children and adolescents admitted to residential care facilities in Japan

**DOI:** 10.1371/journal.pone.0198123

**Published:** 2018-06-01

**Authors:** Masakazu Okada, Masaaki Otaga, Takako Tsutsui, Hisateru Tachimori, Shingo Kitamura, Shigekazu Higuchi, Kazuo Mishima

**Affiliations:** 1 Department of Kansei Science, Graduate School of Integrated Frontier Science, Kyushu University, 4-9-1 Shiobaru, Minami-ku, Fukuoka, Japan; 2 Department of Psychophysiology, National Institute of Mental Health, National Center of Neurology and Psychiatry, 4-1-1 Ogawa-Higashi, Kodaira, Tokyo, Japan; 3 Department of Health and Social Services, National Institute of Public Health, 2-3-6 Minami, Wako-shi, Saitama, Japan; 4 Graduate School of Business, University of Hyogo, 8-2-1 Gakuen-nishimachi, Nishi-ku, Kobe, Japan; 5 National Institute of Mental Health, National Center of Neurology and Psychiatry, 4-1-1 Ogawahigashicho, Kodaira, Tokyo, Japan; 6 Department of Human Science, Faculty of Design, Kyushu University, 4-9-1 Shiobaru, Minami-ku, Fukuoka, Japan; Chiba Daigaku, JAPAN

## Abstract

**Background:**

The psychological care of abused children in the child protection system is an urgent issue in Japan. Child abuse has a serious impact on children’s emotion and behavior, but there is virtually no evidence about how child abuse affects sleep, which is closely related to behavioral and emotional control. In this study, we sought to identify sleep habits and suspected sleep disorders among abused children and adolescents admitted to residential care facilities in Japan and to investigate their association with emotional and behavioral problems.

**Methods:**

The study targeted 273 abused children and adolescents (age range: 4 to 15 years) who had been admitted to a residential care facility in Japan. They were assessed by physicians and other personnel at facilities with expertise in childcare and abuse. Respondents completed a brief sleep questionnaire on the incidence of problematic sleep habits and suspected sleep disorders as well as a questionnaire on emotional and behavioral issues.

**Results:**

Approximately 40% of the abused children and adolescents had some sleep-related symptoms at bedtime and waking, and 19% had suspected sleep disorder. Abused children with emotional and behavioral problems had a significantly higher incidence of suspected sleep disorders than abused children without such problems, and this incidence was particularly high among those with antisocial behavior and depressive behavior. Our predictive model also showed that antisocial behavior and depressive behavior were significant predictors of suspected sleep disorders.

**Conclusion:**

Careful assessment and appropriate therapeutic intervention for sleep disorders are required in abused children and adolescents with emotional and behavioral problems.

## Introduction

Despite the declining population of children in Japan, the number of children receiving child protection services after experiencing maltreatment or abuse has increased. According to a 2017 report by the Ministry of Labour, Health and Welfare, the number of child abuse consultations at the Consultation Office for Children was 103,260 cases in 2015, representing an approximate 3-fold increase over the past 10 years[1]. Because foster care is not common in Japan, the majority of child protection services in Japan are provided in the following 5 types of residential care facilities: (1) foster homes, (2) maternal and child living support facilities, (3) infant homes, (4) short-term therapeutic institutions for emotionally disturbed children, and (5) children's self-reliance support facilities. As of 2017, approximately 45,000 children are in the child protection system, 38,843 of whom (86.5%) have been placed in a facility, with 70.3% of these 38,843 children living in foster homes. The remaining 13.5% of the children in the child protection system receive home-based care, either from a foster parent or in a family home. By 2008, the percentage of previously abused children in foster homes had already reached 53.4%, and the rate of a history of abuse among children in foster homes consistently exceeded 50%[2].

Abuse has serious impacts on children’s mental and physical health. Abuse during childhood can cause a variety of short- and-long term disorders to physical, emotional, and cognitive function[3–5], and cases of severe abuse can increase the risk of mental illness[6,7]. Previous studies have found that emotional and behavioral problems increase among abused children. Child abuse is related to both emotional problems such as depression, suicidal thinking/intention, anxiety[8–10], and behavioral problems such as bullying, tantrums, and delinquency [11,12]. The child protection system for abused children must therefore provide not only protection but also therapeutic care. The disturbance in lifestyle habits and deterioration in mental health that occur shortly after abuse can be important predictors of the long-term effects of abuse on health and quality of life. Nevertheless, therapeutic interventions are limited and evidence-based practices remain scarce[13].

Sleep plays an important role in children’s development and is indispensable in maintaining mental and physical health and functioning[14]. Sleep deprivation and sleep disorders are known to have an adverse impact on emotional, learning, and social functions[15,16]. Moreover, neurodevelopmental and mental disorders such as depression, anxiety disorder, attention deficit hyperactivity disorder (ADHD), and autism spectrum disorder (ASD) are highly prevalent in abused children[7]; these conditions are often accompanied by sleep problems such as insomnia, hypersomnia, and irregular sleep–wake rhythm[17,18]. Conversely, sleep problems in children are both a precursor and a risk factor for the onset and exacerbation of neurodevelopmental and mental disorders[19]. Identifying sleep problems in abused children can therefore help prevent the onset and worsening of mental illness through early detection of emotional and behavioral problems or the risk of their development.

Previous research on abuse and sleep has mostly investigated the effect of childhood abuse over longer time frames[20,21]. More recent longitudinal reports have found that abuse during childhood is associated with sleep issues later in adolescence and into adulthood[22–24]. Meanwhile, the impact of childhood maltreatment on sleep during the ensuing period of infancy and early adolescence is limited. A few studies have suggested that abuse can lead to increased nocturnal activity and reduced sleep efficiency[20,21]. However, there is currently little evidence on the link between sleep disorders and emotional and behavioral problems in abused children. In this study, we sought to identify the problematic sleep habits and sleep disorders of abused children and adolescents admitted to residential care facilities in Japan and to investigate their association with emotional and behavioral problems.

## Methods

### Participants and settings

This study used data drawn from evaluations of protected children and adolescents (ie, emotional and behavioral characteristics and sleep conditions) obtained from a database containing data from a 2008 national survey aimed at understanding the state of care provided in residential care facilities. This database was created as part of the Health and Labour Sciences Research Grant project entitled “Research on the relationship between abuse-induced problems, disorders, and other typified conditions among protected children and the amount of care required”. The target age range for the study was 4 to 15 years old, and the study population consisted of children and adolescents from 2 of the 5 types of residential care facilities: foster homes and maternal and child living support facilities. We excluded infant homes from the scope of our study due to the young age of children at these facilities. We also excluded short-term therapeutic institutions for emotionally disturbed children and children's self-reliance support facilities because they are intended to care for children with severe emotional disorders as well as those with criminal and other antisocial behaviors, so these children have traits different from those at the other types of facilities. Study sites were selected based on adequate staffing and, to eliminate bias in terms of the organizational structure and type of care provided (eg, small group care), by each type of facility. Evaluations were performed and questionnaires were completed by facility personnel, physicians, and nurses with expertise in childcare and abuse, as well as by nursery school teachers, child welfare counselors, and psychotherapists responsible for the children’s care. They were all full-time staff members whose contact with the children was sufficiently frequent and extensive to report sleep, emotional, and behavior problems in daily life.

### Survey description

Using a previously standardized brief 19-item sleep questionnaire for 6- to 12-year-old children (**S1 Table**) [25], we studied the sleep symptoms of participants over the previous month. The survey contained 4 items on bedtime symptoms, 9 items on sleep symptoms, 5 items on waking symptoms, and 1 item on daytime sleepiness symptoms. Respondents evaluated the incidence of each item using a 3-point Likert scale (1 = Rarely [never or once per week], 2 = Sometimes [2 to 4 times per week], and 3 = Usually [5 or more times per week]). Children scoring above the cutoff of 24 points were suspected of having some form of sleep disorder[25]. We presented the percentage of sleep problems based on a subcategory item response of “Usually (5 or more times per week)” or “Sometimes (2–4 times per week).” We also recorded the children’s sleeping habits (bedtime, waking time, midday nap duration), and considered time in bed as the period from bedtime to waking.

Because it was difficult to diagnose neurodevelopmental and mental disorders in the institute, we used the questionnaire of emotional and behavioral problems as a proxy indicator. Children’s emotional and behavioral problems were assessed using the following 5 items: (1) autistic behavior, (2) attachment problems, (3) attention deficit/hyperactive behavior, (4) antisocial behavior, and (5) depressive behavior. Respondents evaluated the incidence of each item using a 4-point scale (1, Not suspected; 2, Possible; 3, Probable; and 4, Definite); children with a rating of 2 or higher were regarded as having the emotional/behavioral problem in question. The score of 2 was selected because we want to detect children not only in the clinical range but also in the borderline range. Each item was evaluated by a physician and by personnel who had undergone training according to our guidebook (**S2 Table**).

Other survey items included experience of abuse (based on information gathered at the time of admission), the type of abuse, and whether the child had a mental disease/disorder according to the physician’s diagnosis. Abuse was classified as follows: (1) physical abuse (assaulting a child in a manner that causes or is likely to cause external physical injury to the child); (2) sexual abuse (engaging in an indecent act against a child or causing the child to engage in an indecent act); (3) neglect (significant failure by a parent or guardian to care for a child); (4) psychological abuse (speech or behavior that causes significant psychological trauma to a child, or domestic violence witnessed by a child); and (5) other abuse. Survey respondents were instructed to select all applicable categories of child abuse (multiple answers permitted).

### Ethical considerations

This study was approved by the Ethics Committee of National Institute of Public Health (NIPH-TRN #08003), Japan and was conducted according to procedures adhering to the ethical guidelines for clinical and epidemiological research. We obtained written informed consent from the legal guardians of minors included in the study.

### Data analysis

All statistical analysis was performed using IBM SPSS Statistics 21.0 (IBM Corporation, Armonk, NY). Comparison of mean values was performed using Student’s t-test, and comparison of frequency for values such as sex was performed using the Chi-square test. The Holm method[26] was used to adjust the P values in multiple testing. We divided sleep parameters into 3 dimensions—3 sleep habits (bedtime, wake time, time in bed), 4 sleep symptoms (bedtime, sleep, waking, and daytime sleepiness symptoms), and suspected sleep disorder—and conducted multiple testing on the first 2 dimensions (3 sleep habits and 4 sleep symptoms) within each row (emotional/behavioral problem). For example, in attachment problems, the multiplicity of the test was adjusted by the Holm method so that the type I error rate of the whole did not exceed 5% in the three items related to sleep habits (bedtime, wake time, time in bed). The same adjustment was also made in the four sleep symptom items. Factors for predicting sleep disorders were determined using binomial logistic regression analysis. Suspected sleep disorder (as indicated by a survey score of ≥ 24) was the dependent variable. Age, sex, duration of admission, type of abuse experienced (physical, sexual, neglect, and psychological), and emotional and behavioral characteristics (attachment problems, autistic behavior, attention deficit/hyperactive behavior, antisocial behavior, and depressive behavior) were the explanatory variables. Stepwise selection (likelihood ratio) was used after confirming that none of the variables had a significant linear relationship on scatter plots.

## Results

### Sample characteristics

The survey population consisted of 332 children and adolescents who had experienced abuse. We excluded 59 children: 35 because they were missing the demographic, emotional, and behavioral items and sleep questionnaire items, and 24 because they were outside of the targeted age range (4 to 15 years old).

Demographic and background characteristics of the remaining 273 children are shown in **Table 1**. Mean age was 9.9 ± 3.2 years, and the sex ratio was almost equal. Mean duration of admission was 4.1 ± 3.0 years. The multiple response question on type of abuse showed that approximately half of the children had experienced neglect (45.4%), psychological abuse (53.1%), and physical abuse (50.5%), whereas 7.7% of children experienced sexual abuse. Children who had experienced only one type of abuse and those who had experienced 2 or more types of abuse comprised 60.1% and 39.9% of respondents, respectively. The most common emotional and behavioral characteristics were antisocial behavior and attention deficit/hyperactive behavior, at approximately 40% each. Depressive behavior was also present in 11.1% of children. Children with 1, 2, or 3 or more emotional and behavioral problems accounted for 21.2%, 17.6%, and 20.5% of the sample, respectively. The age (mean ± SD [range]) of children with each emotional or behavioral characteristic were as follows: attachment problems (yes: 9.7 ± 3.3 [4–15] years; no: 10.6 ± 2.9 [4–15] years); autistic behavior (yes: 9.7 ± 3.3 [4–15] years; no: 11.2 ± 2.9 [6–15] years); attention deficit/hyperactive behavior (yes: 9.8 ± 3.4 [4–15] years; no: 10.1 ± 2.9 [4–15] years); antisocial behavior (yes: 9.4 ± 3.3 [4–15] years; no: 10.8 ± 2.9 [4–15] years); and depressive behavior (yes: 9.6 ± 3.2 [4–15] years; no: 12.3 ± 2.2 [9–15] years). Among children diagnosed with a mental disorder, 18.3% had a neurodevelopmental disorder, 9.5% had a behavioral disorder, 8.4% had an anxiety disorder, and 1.8% had a mood disorder. Abused children with no emotional or behavioral problems accounted for 39.6% of the sample.

**Table 1:** Demographic and background characteristics of abused children.

	N = 273
Sex (male/female)	140/133
Age (years)	9.9 (3.2)
Admission period (years)	4.1 (3.0)
Type of abuse, n (%)	
Physical	124 (45.4)
Sexual	21 (7.7)
Neglect	145 (53.1)
Psychological	138 (50.5)
Other	2 (0.7)
Mental disorder, n (%)	60/267 (22.5)
Emotional/behavioral characteristics, n (%)	
Attachment problems	62/269 (23.0)
Autistic behavior	37/270 (13.7)
Attention deficit/hyperactive behavior	103/269 (38.3)
Antisocial behavior	108/269 (40.1)
Depressive behavior	30/270 (11.1)

Continuous variables are given as mean (SD). Prevalence is reported as a percentage.

### Sleep problems (sleep habits and suspected sleep disorders)

**Table 2** shows the sleep habits (bedtime, waking time, mean time in bed, midday nap duration) and the percentage of children with each type of symptom (bedtime, sleeping, waking, and daytime sleepiness) as determined by the brief sleep questionnaire. Approximately 40% of children had bedtime and waking symptoms, while nearly 20% of children had sleeping symptoms and roughly 10% had daytime sleepiness. The percentage of children with some form of suspected sleep disorder (survey score ≥ 24 points) was 19.4%. The most commonly selected item in the brief sleep questionnaire was “Difficulty getting out of bed in the morning” (35.2%), while symptoms with a response rate of ≥ 15% were “Poor waking habits” (26.0%), “Fear of sleeping in the dark” (23.4%), “Wakes up in a negative mood” (19.8%), “Resists going to bed at bedtime” (19.0%), and “Needs special object to fall asleep” (16.5%) (**S3 Table**).

**Table 2:** Sleep habits and incidence of sleep-related symptoms and sleep disorders.

Sleep habits (h)	N = 273
Bedtime	21.2 (1.0)
Waking time	6.6 (0.8)
Time in bed	9.4 (1.2)
Daytime nap	0.2 (0.6)
Sleep problems, n (%)	N = 273
Bedtime symptoms	117 (42.9)
Sleep symptoms	50 (18.3)
Waking symptoms	99 (36.3)
Daytime sleepiness symptoms	27 (9.9)
Sleep disorder, n (%)	N = 206
Suspected sleep disordered children	42 (20.4)

Continuous variables are given as mean (SD). Prevalence is reported as a percentage.

### Emotional and behavioral problems and sleep

We investigated the relationship between emotional and behavioral problems and sleep in abused children by comparing the percentage of children with various sleep habits, sleep-related symptoms, and suspected sleep disorders according to presence or absence of emotional and behavioral problems (**Table 3**). We found that the presence or absence of emotional and behavioral problems did not cause a major difference in sleep habits in children receiving lifestyle guidance in a care facility. However, children with autistic behavior spent a significantly shorter time in bed than children without such behavior (9.0 ± 1.3 vs 9.5 ± 1.2, p = 0.02). Moreover, children with attention deficit/hyperactive behavior went to bed significantly earlier (21.0 ± 0.9 vs 21.3 ± 1.0, p = 0.01) and children with depressive behavior went to bed significantly later (21.8 ± 1.2 vs 21.1 ± 0.9, P < 0.01) and woke up significantly later (7.2 ± 1.8 vs 6.5 ± 0.6, P < 0.01) than their counterparts without such behaviors.

**Table 3:** Sleep habits and incidence of sleep-related symptoms and sleep disorders by presence/absence of emotional and behavioral problems in abused children.

		Sleep habits			Sleep symptoms				Suspected sleep disorder (%)
		Bedtime (h)	Wake time (h)	Time in bed (h)	Bedtime symptoms (%)	Sleep symptoms (%)	Waking symptoms (%)	Daytime sleepiness Symptoms (%)	
Attachment problems	Yes	21.1 (1.1)	6.7 (1.2)	9.7 (1.7)	53.2	24.2	41.9	9.7	19.6
	No	21.3 (1.0)	6.6 (0.7)	9.3 (1.0)	40.1	16.9	34.8	9.7	20.8
	ES	.10	.11	.15	.13	.08	.06	.00	-.01
	P	0.184	0.186	0.030	0.080	0.198	0.367	1.000	1.000
Autistic behavior	Yes	21.5 (0.7)	6.4 (1.2)	9.0 (1.3)	45.9	21.6	37.8	13.5	25.9
	No	21.2 (1.0)	6.6 (0.8)	9.5 (1.2)	42.5	18.0	36.1	9.0	19.7
	ES	.08	.08	.14	.04	.03	.01	.05	.05
	P	0.102	0.075	0.019	0.723	0.649	0.855	0.373	0.449
Attention deficit/hyperactive behavior	Yes	21.0 (0.9)	6.5 (0.8)	9.5 (1.1)	50.5	22.3	39.8	7.8	25.3
	No	21.3 (1.0)	6.7 (0.8)	9.4 (1.2)	38.6	16.3	33.7	10.2	17.6
	ES	.16	.12	.05	.17	.08	.06	.00	-.01
	P	0.012^a^	0.054	0.412	0.059	0.259	0.361	0.666	0.215
Antisocial behavior	Yes	21.2 (1.1)	6.6 (1.1)	9.4 (1.6)	50.9	25.0	38.0	13.0	29.3
	No	21.2 (0.9)	6.6 (0.5)	9.4 (0.9)	37.3	14.3	34.8	7.5	13.9
	ES	.02	.05	.02	.16	.14	.03	.09	.19
	P	0.804	0.398	0.773	0.033	0.037	0.607	0.145	0.012
Depressive behavior	Yes	21.8 (1.2)	7.2 (1.8)	9.4 (2.4)	60.0	16.7	53.3	20.0	45.0
	No	21.1 (0.9)	6.5 (0.6)	9.4 (1.0)	40.8	18.8	34.2	8.3	17.8
	ES	.22	.24	.02	.15	-.02	.13	.12	.20
	P	0.000^a^	0.000^a^	0.868	0.052	1.000	0.045	0.052	0.008

Continuous variables are described as mean (SD). ES = effect size.

Effect sizes are denoted as *d* for the t-test and *φ* for the Chi-squared test.

Continuous variables and categorical variables were tested using the unpaired t-test and Chi-squared test, respectively.

^a^ Statistically significant after adjustment for multiple tests using the Holm method for 2 dimensions (3 sleep habits and 4 sleep symptoms) within each row (each emotional/behavioral problem).

All of the abused children with emotional and behavioral problems, except those with autistic behavior, had more frequent sleep-related symptoms compared with the abused children without emotional and behavioral problems—particularly bedtime symptoms such as resisting going to bed and fear of sleeping in the dark. Specifically, children with antisocial behavior had a significantly higher incidence of bedtime and sleeping symptoms, and those with depressive behavior had a significantly higher incidence of sleeping and waking symptoms than their counterparts. As a result, children with antisocial behavior and depressive behavior presented with a significantly higher frequency of suspected sleep disorder than their counterparts.

Conversely, a comparison of the percentage of emotional and behavioral problems among children in the suspected sleep disorder group and the non-suspected group revealed that the percentage of children with antisocial behavior and depressive behavior was significantly higher in the suspected sleep disorder group (**Table 4**).

**Table 4:** Incidence of emotional and behavioral problems by presence/absence of suspected sleep disorder in abused children.

	Suspected sleep disorder (%) (N = 42)	No suspected sleep disorder (%) (N = 164)	P
Autistic behavior	16.7	12.3	0.449
Attachment problems	21.4	22.7	1.000
Attention deficit/hyperactive behavior	47.6	36.4	0.215
Antisocial behavior	58.5	35.6	0.012*
Depressive behavior	21.4	6.7	0.008*

* p<0.05 by the Chi-squared test

### Predictive model of sleep disorders

Binomial logistic regression analysis revealed the predictive factors of sleep disorder were young age (odds ratio [OR], 0.87; 95% confidence interval [CI], 0.77–0.99, p = 0.036), antisocial behavior (OR, 2.82; 95% CI, 1.32–6.07, p = 0.008), and depressive behavior (OR, 4.43; 95% CI, 1.46–13.4, p = 0.009). The result of the Chi-square test to detect the likelihood ratio of the model of sleep disorders compared with the model including all factors was significant at p = 0.001, while that of the Hosmer–Lemeshow test was p = 0.972, and the percentage of correct classifications was 78.9%.

## Discussion

In this study, we investigated the association between problematic sleep habits and incidence of suspected sleep disorders, and emotional and behavioral problems among abused children and adolescents under the protection of residential care facilities in Japan.

Previous research has found that emotional and behavioral problems are more likely among abused children. Childhood abuse is linked to emotional problems such as depression, suicidal thoughts/intent, and anxiety[8–10], as well as behavioral problems in the form of bullying, tantrums, and delinquency[11,12]. Antisocial behavior may be associated with neurodevelopmental disorders such as ADHD and ASD, and may also be triggered by environmental factors such as attachment problems[27]. The global estimated prevalence of ADHD was reported as 6.7%-7.8%[28], and the prevalence of major depressive disorder (MDD) in Japanese community adolescents was reported as 4.9% (2.2% in boys and 8.0% in girls)[29]. Another study examining the behavioral and emotional problems using the “Strengths and Difficulties Questionnaire”[30] suggested that conduct problems (14.3%) and hyperactivity/inattentiveness (16.5%) exceeding the borderline range in non-abused Japanese children (aged 4–12 years)[31]. Compared with these results, the abused children in the present study had a higher incidence of emotional and behavioral problems; antisocial behavior were seen in 40.1%, attention deficit/hyperactive behavior in 38.3%, and depressive behavior in 11.1% of respondents.

The prevalence of developmental and other mental disorders among abused children in residential care facilities in Europe is 19%-23% for MDD, 10%-32% for ADHD, and 23%-26% for ASD[7,32,33]. In the U.S. and Canada, the prevalence of mental disorders among abused children in foster care is 32%-44%, with 10%-38% for ADHD and 5%-32% for depression[34–37]. Thus, there are no substantial differences in the rates of emotional and behavioral problems between abused children receiving child protection services in Western countries and the abused children in this study.

We also found that the abused children had various sleep-related symptoms in terms of sleep onset latency, duration of sleep, and waking time. When comparing the rates obtained for each symptom on the 19-item brief sleep questionnaire with the data for non-abused children in the aforementioned Health and Labour Sciences Research Grant project[38], we found that the abused children tended to have more symptoms, specifically the bedtime symptom of fear of sleeping in the dark (23.4% vs 13.9%), the sleeping symptoms of night terrors (1.8% vs 0.5%) and nightmares (4.4% vs 1.7%), the waking symptoms of poor waking habits (26.0% vs 20.5%) and early awakening (3.7% vs 1.9%), and the daytime symptom of sleepiness (9.9% vs 6.9%) (**S3 Table**). In particular, the abused children with emotional and behavioral problems had a higher rate of suspected sleep disorders than the abused children who had no emotional and behavioral problems. Our predictive model also showed that antisocial behavior and depressive behavior were significant predictors of sleep disorders. The children with suspected sleep disorders had a higher incidence of emotional and behavioral problems than their counterparts, and 58.8% of these children displayed antisocial behavior, while 47.6% had attention deficit/hyperactive behavior and 21.4% had depressive behavior.

Previous studies have also demonstrated that sleep disorders occur not only in children who have experienced abuse, but also in children with mental disorders. For instance, disorders of initiating and maintaining sleep and excessive daytime sleepiness often occur in children with ADHD and depression, and these conditions are associated with a range of sleep disorders such as insomnia, circadian rhythm sleep–wake disorder (delayed sleep-phase disorder), sleep-related breathing disorder, restless legs syndrome, and periodic limb movement disorder[17,18]. In addition, fear of sleeping in the dark, night terrors, and nightmares often occur in children with developmental disorders[18], while poor waking habits, early awakening, and daytime sleepiness are closely related to circadian rhythm sleep–wake disorder and insomnia[37]. This suggests that children with these symptoms require special monitoring. The abused children with emotional and behavioral problems in the present study all had a high incidence of bedtime symptoms such as resisting going to bed and fear of sleeping in the dark, regardless of the nature of their problems. Abused children with antisocial behavior and depressive behavior also had difficulty with sleeping and waking. Because the delay in bedtime and waking time was seen even in environments where guidance on sleep hygiene is provided, it is reasonable to interpret that these variations in the sleep habits of abused children with emotional and behavioral problems were attributable to sleep disorders. The findings of this study suggest that abused children with emotional and behavioral problems may have sleep disorders that cannot be resolved simply by adjusting their sleep habits, and that appropriate therapeutic intervention is needed to adjust their schooling and lifestyle patterns. Both behavioral therapy and pharmacological interventions are recommended due to their demonstrated efficacy in treating pediatric sleep disorders[39].

Research on the association between childhood maltreatment and sleep during the ensuing period of infancy and adolescence is limited, but previous studies have suggested that abuse can lead to increased nocturnal activity and reduced sleep efficiency[20,21]. It has also been reported that more severe abuse during childhood is related to increased sleep disturbances during adolescence, and that such abuse contributes to sleep disorders by inducing psychological distress[40]. Moreover, sleep typically takes place when a person feels safe and secure, but the tendency to experience such feelings may be compromised in children with a history of maltreatment. A number of studies have asserted that childhood sleep disorders are closely linked to emotional and behavioral problems, and that both internalized and externalized disorders are correlated with sleep disorders[41]. Moreover, therapeutic interventions in children with sleep disorders have also proven effective in improving symptoms of mental illness such as ADHD and depression[42–44]. Our study was based on a cross-sectional survey, and so we could not demonstrate a causal relationship between sleep habits and sleep disorders and the onset and exacerbation of emotional and behavioral problems. Nonetheless, our findings suggest that sleep disorders can affect the emotional and behavioral problems of abused children. Further research is needed to determine how therapeutic interventions for sleep disorders affect the emotional and behavioral problems and outcomes of abused children.

In this study, however, problematic sleeping habits were minimal among the abused children. Compared with the children who had no emotional or behavioral problems, the children with these problems had a tendency toward later bedtime and waking time, but these differences were small. One possible reason for this behavior is that Japan’s residential care facilities are mandated by national policy to promote a domestic form of childcare, to enable same-aged children and facility staff to have a close-knit and well-regulated group lifestyle. Within these facilities, children in each age group adhere to a time schedule for waking, going to bed, bathing, eating, and studying. According to the Health and Labour Sciences Research Grant project entitled “Prevalence of developmental disorders and developmental changes in children around schooling age: A cross-sectional and longitudinal community-based study”, which targeted 25,211 typical elementary school students in 10 communities in Japan and used the same brief sleep questionnaire as we used in the present study, children living in the community (mean age of 10.1 ± 2.7 years) had a bedtime at 21.9 ± 1.0 h, a waking time of 6.6 ± 0.7 h, and a mean time in bed of 8.8 ± 1.2 h (in Japanese). Compared with these data, the abused children in our study went to bed approximately 40 min later, and their time in bed was approximately 40 min longer. Furthermore, the sleeping habits of facility-based children in our study indicated that they had an adequate time in bed compared with the average sleep time of 10 year olds in various countries as reported by Olds et al.[45]. Additionally, their sleep time of 9–11 h is in line with that recommended for 6 to 13 year olds by the American National Sleep Foundation[46]. The above findings suggest that the children in our study might have more sleep problems despite keeping a relatively good sleeping schedule.

We recognize several limitations in this study. First, time in bed is merely that, and it is not an accurate indicator of sleep quality such as sleep duration and wake after sleep onset. Previous studies that used actigraphy to monitor the sleep state of abused children reported increased nocturnal activity and reduced sleep efficiency, indicating that abused children may suffer from diminished sleep quality and insomnia. Second, residential care facilities in Japan provide a relatively high level of care, restricting our ability to make generalizations about the results with regards to abused children living in other environments. Abused children who are not in protective care may also include those with more serious mental health issues and sleep disorders. Third, the size and workforce of each facility in this study varied, and this variation may have affected the accuracy of evaluating children’s sleep states and sleep habits by facility personnel. Fourth, we did not obtain control group data from children living in the community, and thus further research is required to compare the findings of abused children in facilities with those of non-abused children. Fifth, it was not our intention in this study to undertake a detailed investigation of mental illnesses such as developmental disorders and depression; consequently, the accuracy of our diagnoses of emotional and behavioral problems may not be consistent with those of other studies. We are therefore not able to make unequivocal comparisons with the respective prevalence rates in countries outside Japan. Nevertheless, our findings did not depart substantially from the results of surveys conducted on abused children in residential care facilities in Western countries. Finally, the items on children’s emotional and behavioral problems do not clearly define the five selected neurodevelopmental and psychiatric disorders, and do not indicate how the definitions of these disorders specifically relate to their measurement of emotional and behavioral problems. Each “condition” or “problem” is multi-dimensional in nature, and the number of items in the measure does not capture this. The items themselves are also not exclusive to an individual disorder, but instead cut across them (eg, *sexual problems* or *argues and bullies with other children* within the area of antisocial behavior). Though not used in this study, available standardized measures such as the Child Behavior Checklist (CBCL)[47] contain well-described constructs of emotional and behavioral problems and could be used in future studies. Moreover, when correcting for multiple comparisons using the Hosmer–Lemeshow test, their predictive model was non-significant. Hence the results must be interpreted with caution.

Despite these limitations, our study targeted a large number of children compared with previous studies investigating the association between abuse and sleep. Abused children who are under the protection of residential care programs require treatment for their mental and physical disorders. The disturbance of lifestyle habits and deterioration of mental health that occur shortly after abuse can be important predictors of the long-term effects of abuse on health and quality of life. Nevertheless, therapeutic approaches are limited, and evidence-based practices remain scarce. In this study, we demonstrated that abused children who have emotional and behavioral problems also require treatment for sleep disorders, especially when they have sleep symptoms such as “fear of sleeping in the dark”, “night terrors”, “nightmares”, and “early awakening”. Thus, the present study contributes toward the goal of early detection and intervention for abused children who require treatment.

## Conclusion

In this study, we identified abused children and adolescents with some form of sleep symptoms or suspected sleep disorders. We also showed that abused children with emotional and behavioral problems manifesting as antisocial behavior and depressive behavior had a significantly high incidence of suspected sleep disorders. Abused children and adolescents with emotional and behavioral problems need to be evaluated for sleep disorders and should receive therapeutic intervention that goes beyond instruction on sleep hygiene.

## Supporting information

S1 Table Brief sleep questionnaire (19 items). Please answer the following questions based on your recollection of how the child has slept over the past month. Describe the child’s sleeping habits on normal days rather than on days when something out of the ordinary occurred (eg, when the child caught a cold).
(DOCX)

S2 Table Procedure for evaluating emotional and behavioral problems. (DOCX)

S3 Table Prevalence of sleep symptoms in abused children. (DOCX)
