# Supplementary material for: Association of sleep with emotional and behavioral problems among abused children and adolescents admitted to residential care facilities in Japan
Source: PLoS One. 2018 Jun 1;13(6):e0198123. doi: 10.1371/journal.pone.0198123 (PMC5983560; doi:10.1371/journal.pone.0198123)
Supplement: S1 Table — Please answer the following questions based on your recollection of how the child has slept over the past month. Describe the child’s sleeping habits on normal days rather than on days when something out of the ordinary occurred (eg, when the child caught a cold). (DOCX) [file pone.0198123.s001.docx]

**S1 Table. Brief sleep questionnaire (19 items)**

Please answer the following questions based on your recollection of how the child has slept over the past month.

Describe the child’s sleeping habits on normal days rather than on days when something out of the ordinary occurred (eg, when the child caught a cold).

|  |  | Almost always (5-7 times/week) | Sometimes  (2-4 times/week) | Rarely  (no more than 1 time/week) |
| --- | --- | --- | --- | --- |
| **Bedtime symptoms** | |  |  |  |
|  | Child falls asleep with rocking or rhythmic movements | □ | □ | □ |
|  | Child needs special object to fall asleep  (doll, special blanket, etc.) | □ | □ | □ |
|  | Child resists going to bed at bedtime | □ | □ | □ |
|  | Child is afraid of sleeping in the dark | □ | □ | □ |
| **Sleep symptoms** | |  |  |  |
|  | Child talks during sleep | □ | □ | □ |
|  | Child is restless and moves a lot during sleep | □ | □ | □ |
|  | Child sleepwalks during the night | □ | □ | □ |
|  | Child grinds teeth during sleep  (your dentist may have told you this) | □ | □ | □ |
|  | Child snores loudly | □ | □ | □ |
|  | Child appears to stop breathing during sleep | □ | □ | □ |
|  | Child snorts and/or gasps during sleep | □ | □ | □ |
|  | Child awakens during night screaming, sweating, and inconsolable | □ | □ | □ |
|  | Child awakens alarmed by a frightening dream | □ | □ | □ |
| **Waking symptoms** | |  |  |  |
|  | Child wakes up in negative mood | □ | □ | □ |
|  | Child has difficulty getting out of bed in the morning | □ | □ | □ |
|  | Child takes a long time to become alert in the morning | □ | □ | □ |
|  | Child wakes up very early in the morning | □ | □ | □ |
|  | Child has a good appetite in the morning | □ | □ | □ |
| **Daytime** sleepiness **symptoms** | |  |  |  |
|  | Child suddenly falls asleep in the middle of active behavior | □ | □ | □ |
