# Supplementary material for: Association of sleep with emotional and behavioral problems among abused children and adolescents admitted to residential care facilities in Japan
Source: PLoS One. 2018 Jun 1;13(6):e0198123. doi: 10.1371/journal.pone.0198123 (PMC5983560; doi:10.1371/journal.pone.0198123)
Supplement: S2 Table — (DOCX) [file pone.0198123.s002.docx]

**S2 Table. Procedure for evaluating emotional and behavioral problems**

1. Autistic behavior
   - Observe and evaluate the child for the following:
     - No noticeable delay in speech but occasional inability to express feelings when communicating with others
     - Continually focuses on one thing without any variability
     - Has great difficulty encountering new places/new experiences and is unable to adapt
2. Attachment problems
   - Please evaluate whether the child exhibits the following two behaviors:
     - Constantly cautious and anxious towards carers and resists or refuses (occasionally in a violent manner) to be touched (symptoms of inhibited attachment disorder)
     - Is excessively friendly with anyone including strangers, and engages in excessive contact (symptoms of disinhibitive attachment disorder)
3. Attention deficit/hyperactive behavior
   - Please evaluate whether the child frequently exhibits the following behaviors:
     - Easily distracted and unable to concentrate (inattentiveness)
     - Restless and unable to remain still for prolonged periods
     - Constantly fidgeting and shifting about
4. Antisocial behavior
   - Please evaluate whether the child frequently exhibits the following behaviors:
     - Loses temper and throws tantrums
     - Argues with and bullies other children
     - Lies and deceives
     - Sexual problems, stays out without permission
     - Engages in theft and arson
5. Depressive behavior
   - Please evaluate whether the child continually exhibits any of the following behaviors for at least 1 to 2 weeks:
     - Irritable and moody almost the entire day
     - Does not enjoy meals and has no appetite
     - Makes statements about life not being worth living and wanting to die, etc.
