# Supplementary material for: Association of sleep with emotional and behavioral problems among abused children and adolescents admitted to residential care facilities in Japan
Source: PLoS One. 2018 Jun 1;13(6):e0198123. doi: 10.1371/journal.pone.0198123 (PMC5983560; doi:10.1371/journal.pone.0198123)
Supplement: S3 Table — (DOCX) [file pone.0198123.s003.docx]

**S3 Table Prevalence of sleep symptoms in abused children**

|  | Community children (%)  (N=26,903) | Abused children (%)  (N=273) |
| --- | --- | --- |
| Resists going to bed | 17.4 | 19.0 |
| Falls asleep with rhythmic movements | 1.4 | 2.6 |
| Needs special object to fall asleep | 14.1 | 16.5 |
| *Afraid of sleeping in the dark | 13.9 | 23.4 |
| *Talks during sleep | 16.1 | 13.2 |
| Moves a lot during sleep | 8.0 | 9.5 |
| Sleepwalks | 0.7 | 1.1 |
| *Grinds teeth | 12.8 | 7.3 |
| Snores | 8.6 | 5.1 |
| Stops breathing | 1.3 | 0.7 |
| Snorts/gasps | 1.1 | 0.7 |
| Night terrors | 0.5 | 1.8 |
| Nightmares | 1.7 | 4.4 |
| Wakes up in negative mood | 20.1 | 19.8 |
| *Difficulty getting out of bed | 32.0 | 35.2 |
| Poor waking habits | 20.5 | 26.0 |
| Wakes up early | 1.9 | 3.7 |
| *No appetite | 8.8 | 8.1 |
| Daytime sleepiness | 6.9 | 9.9 |
| *Suddenly falls asleep | 0.4 | 0.7 |

Asterisk (*) denotes school-age children only.
